# Supplementary material for: The Downregulation of the Liver Lipid Metabolism Induced by Hypothyroidism in Male Mice: Metabolic Flexibility Favors Compensatory Mechanisms in White Adipose Tissue
Source: Int J Mol Sci. 2024 Oct 8;25(19):10792. doi: 10.3390/ijms251910792 (PMC11477049; doi:10.3390/ijms251910792)
Supplement: Supplementary file 1 [file ijms-25-10792-s001.zip › ijms-3144374-supplementary.pdf]

S1

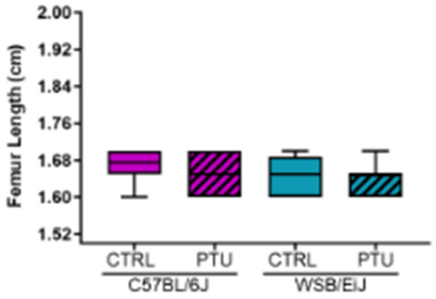

Figure S1: Peripheral hypothyroidism assessment after PTU treatment

Table S1: TaqMan gene expression assays used for qPCR analysis (related to Figure 2 and Figures 4-6)

Gene name	Association	TaqMan Gene expression assay ID
Melanocortin 4 receptor (*Mc4r*)	Central and peripheral metabolic genes (lipid metabolism, food intake, energy metabolism)	Mm00457483_s1
Proopiomelanocortin (*Pomc*)		Mm00435874_m1
Agouti related neuropeptide (*Agrp*)		Mm00475829_g1
Leptin receptor (*Lepr*)		Mm00440181_m1
Peroxisome proliferator activated receptor alpha (*Pparα*)		Mm00440939_m1
Peroxisome proliferator activated receptor gamma (*Pparγ*)		Mm01184322_m1
Patatin-like phospholipase domain containing 2 (*Pnpla2*)		Mm00503040_m1
Peroxisome proliferative activated receptor, gamma, coactivator 1 alpha (*Ppargc1α*)		Mm01208835_m1
Fibroblast growth factor 21 (*Fgf21*)		Mm00840165_g1
Fatty acid synthase (*Fasn*)		Mm00662319_m1
Acetyl-Coenzyme A carboxylase alpha (Acacα)		Mm01304277_m1
Leptin (*Lep*)		Mm00434759_m1
Interleukin 1 beta (*Il1β*)	Inflammatory genes (cytokines, inflammatory pathway)	Mm00434228_m1
Interleukin 10 (*Il10*)		Mm00439614_m1
Interleukin 6 (*Il6*)		Mm00446190_m1
Tumor necrosis factor (*Tnfα*)		Mm00443258_m1
Hypoxanthine guanine phosphoribosyl transferase (*Hprt*)	Housekeeping genes	Mm00446968_m1
Actin beta (*Actb*)		Mm00607939_s1
Eukaryotic translation initiation factor 2A (*Eif2a*)		Mm01289723_m1
Beta-2-microglobulin (*B2m*)		Mm00437762_m1
Phosphoglycerate kinase 1 (*Pgk1*)		Mm00435617_m1
Tyrosine 3-monooxygenase/tryptophan 5-monooxygenase activation protein, zeta polypeptide (*Ywhaz*)		Mm03950126_s1

Table S2: Summary table of the statistics (*p*-value) for metabolic (related to Figure 1 to Figure 5) and inflammatory parameters (related to Figure 6-7). Statistical non-parametric two-way ANOVA with permutations tests (strain; treatment; Strain*treatment effects) were performed followed by post-hoc analysis (indicated on each graph). nqs= not quite significant; ns= not significant

Parameter		Strain	Treatment	Strain*Treatment
			p-value	
**Body Fat mass (Figure 1)**	eWAT weight	0.0001	0.02	ns
	Leptin	0.00039	ns	< 0.00001
**Hypothalamic energy balance (Figure 2)**	*Agrp*	0.033	0.00019	ns
	Pomc	ns	0.0073	ns
	Lepr	0.017	0.038	ns
	Mc4r	ns	ns	0.006
**Circulating lipids (Figure 3)**	Triglycerides	ns	ns	0.025
	Cholesterol	0.045	0.00019	0.031
	HDL	0.0033	0.00019	ns
	LDL	ns	0.00019	0.01
	NEFA	0.012	0.017	0.024
**Hepatic lipid metabolism (Figure 4)**	*Chrebp*	ns	0.00019	ns
	Ppargc1α	ns	0.0125	0.047
	Acacα	ns	0.0003	ns
	Fasn	ns	0.00019	0.045
	Pparα	ns	0.00019	ns
	Fgf21	0.019	ns	0.004
**Adipose lipid metabolism (Figure 5)**	*Acacα*	ns	ns	0.011
	Fasn	ns	0.025	0.007
	Ppar γ	0.00019	ns	ns
	Ppargc1α	0.00039	0.00019	0.022
	Pnpla2	ns	nqs=0.059	ns
**Inflammatory markers (Figure 6)**	*Il10*	ns	0.00059	ns
	Il1β	nqs=0.056	0.03	< 0.00001
	Tnfα	0.045	0.00019	ns
	IFNγ	0.0021	ns	ns
	IL10	0.00019	ns	0.044
	IL1β	ns	ns	0.003
**Hypothalamic glial cells (Figure 7)**	GFAP	0.00019	ns	ns
	IBA1	ns	0.034	0.048
